# The Association between Herpes Zoster and Increased Cancer Risk: A Nationwide Population-Based Matched Control Study

**DOI:** 10.3390/curroncol28040237

**Published:** 2021-07-17

**Authors:** Ji-Hoon Sim, Hyun-Seok Cho, Young-Do Kim, Juhan Mun, Sung-Bae Kim, Jong-Hyuk Lee, Jeong-Gil Leem

**Affiliations:** 1 Department of Anesthesiology and Pain Medicine, Asan Medical Center, University of Ulsan College of Medicine, Seoul 05505, Korea; atlassjh@hanmail.net (J.-H.S.); chohs@amc.seoul.kr (H.-S.C.); midorimd@naver.com (Y.-D.K.); bk3605@naver.com (J.M.); 2 Department of Oncology, Asan Medical Center, University of Ulsan College of Medicine, Seoul 05505, Korea; sbkim3@amc.seoul.kr

**Keywords:** cancer risk, herpes zoster, postherpetic neuralgia, hazard ratio, lymphoid cancer

## Abstract

Background: Herpes zoster (HZ) is strongly associated with decreased immune function, a factor of cancer development. Previous studies suggested inconsistent results regarding the association between HZ and increased cancer risk. We aimed to analyze the association between HZ and specific cancer risk. Methods: Of 134,454 patients diagnosed with HZ between 2002 and 2015, 81,993 HZ patients were matched 1:1 with non-HZ individuals by age, sex, and Charlson comorbidity index. Both groups were examined at 1, 3, and 5 years for cancer diagnosis. A Cox proportional hazard regression model was used to estimate cancer risk in both groups. The postherpetic neuralgia (PHN) and non-HZ groups were compared for specific cancer risk. Results: The HZ group showed a slightly decreased overall cancer risk compared with the non-HZ group (hazard ratio [HR] 0.94, 95% confidence interval [CI] 0.90–0.97, *p* = 0.002). The HRs for specific cancer risk were 0.41 (95% CI, 0.33–0.50, *p* < 0.001); 0.86 (95% CI, 0.81–0.91, *p* < 0.001); 0.87 (95% CI, 0.78–0.97, *p* = 0.014); 0.80 (95% CI 0.73–0.87, *p* < 0.001); 1.20 (95% CI, 1.07–1.34, *p* = 0.001); and 1.66 (95% CI, 1.35–2.03, *p* < 0.001) for cancers of the lips, mouth, and pharynx; digestive system; respiratory system; unknown secondary and unspecified sites; thyroid and endocrine glands; and lymphoid and hematopoietic systems, respectively. The HZ with PHN group showed higher HR for specific cancer risk, such as lymphoid and hematopoietic systems (95% CI, 1.27–2.39, *p* < 0.001). Conclusion: HZ was associated with increased or decreased incidence of specific cancers. PHN further increased the risk of developing certain cancers in HZ patients.

## 1. Introduction

Herpes zoster (HZ) is a viral disease that occurs due to the reactivation of varicella-zoster virus (VZV) when a person’s immunity decreases; it is accompanied by a painful skin rash with blisters [1]. HZ has an incidence of 1.2–4.8 per 1000 individuals, an estimated annual incidence of ≥0.20% in the population, and occurs mainly in the elderly [2,3,4,5]. HZ is strongly associated with decreased immune function [6,7], which is a factor involved in the development of such cancers as melanoma and bladder cancer. These cancers are more likely to occur in patients infected with the human immunodeficiency virus (HIV) and transplant recipients [8,9]. Several previous studies have suggested an association between HZ and increased risk of cancer, but with inconsistent results; therefore, this association remains controversial [10,11,12,13,14,15,16,17,18,19,20,21,22,23,24]. In 1984, Fueyo and Lookingbill reported that HZ was not a marker of internal malignancy; however, the study sample size was too small [20]. In 2005, Buntinx et al. reported that among HZ patients, women and patients aged >65 years had a higher incidence of cancer [23]. In 2011, Ho et al. [19] reported that HZ ophthalmicus may be a marker for increased risk of cancer. However, these studies did not adjust for confounding factors, such as patients’ comorbidities [19,23]. Therefore, we conducted a nationwide population-based matched (by age, sex, and Charlson comorbidity index [CCI]) cohort study, using the Korean National Health Insurance Services (NHIS) data to investigate the association between HZ and cancer.

## 2. Materials and Methods

### 2.1. Study Design and Population

This study was approved by the institutional review board (IRB) of Asan Medical Center (protocol number 2017-0498), and the requirement for written informed consent was waived by the IRB. Our retrospective cohort study was performed by investigating the NHIS-National Sample Cohort claims in South Korea, which comprised complete medical records, including sociodemographic variables, diagnostic codes, deaths, drug utilization review, and medical fee verification [25,26,27].

**Figure 1:**
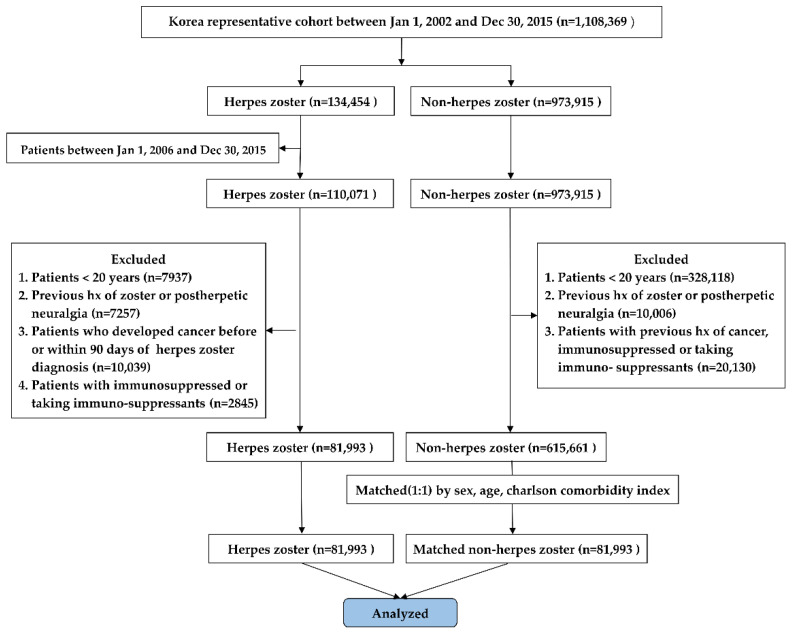
Study flow chart.

The cohort comprised 1,108,369 patients, of which 134,454 patients were diagnosed with HZ between 1 January 2002 and 30 December 2015. The wash-out period was set from 1 January 2002 to 31 December 2005. Therefore, patients presenting with HZ between 1 January 2006 and 30 December 2015 were included in this study (n = 110,071). The exclusion criteria were patients aged <20 years (n = 7937), who had previous history of HZ or postherpetic neuralgia (PHN) (n = 7257), who developed cancer before or within 90 days of HZ diagnosis (n = 10,039), and who were immunosuppressed (organ transplant surgery, chronic renal failure, and chronic liver failure) or taking immunosuppressants (n = 2845). A total of 81,993 HZ patients were enrolled in the HZ group (Figure 1).

Of the 1,108,369 patients, 973,915, who were not diagnosed with HZ between 1 January 2002 and 30 December 2015, were considered as controls. Of these, 358,254 patients met the exclusion criteria and a total of 615,661 patients were enrolled into the non-HZ group (Figure 1). Finally, we performed a 1:1 matching by age, sex, and CCI, between the HZ and non-HZ groups (Figure 1). CCI is a representative method of categorizing the comorbidities of patients based on the International Classification of Diseases (ICD) diagnosis codes [28].

### 2.2. Identification of Herpes Zoster, Cancer, and Confounding Factors

Among the total 1,108,369 patients enrolled in the database, we identified those with first-ever diagnosis of HZ and first-ever diagnosis of cancer using the relevant diagnostic codes of the ICD 10th revision (ICD-10). HZ was classified as ICD-10 code B02, while PHN was classified as “other postherpetic nervous system involvement” (ICD-10 code B02.29). For the subgroup analysis, specific cancers were classified as malignant neoplasms of the following: lips, mouth, and pharynx (ICD-10 codes C00–C14); digestive system (ICD-10 codes C15–C26); respiratory and intrathoracic organs (ICD-10 codes C30–C39); bone and articular cartilage (ICD-10 codes C40–C41); melanoma and other malignant neoplasms of the skin (ICD-10 codes C43–C44); mesothelial and soft tissue (ICD-10 codes C45–C49); urinary tract (ICD-10 codes C64–C68); eye, brain, and other parts of the central nervous system (ICD-10 codes C69–C72); thyroid gland and other endocrine glands (ICD-10 codes C73–C75); unknown, secondary, and unspecified sites (ICD-10 codes C76–C80); and lymphoid, hematopoietic, and related tissues (ICD-10 codes C81–C96). Confounding factors that may affect the development of cancer, such as myocardial infarction, congestive heart failure, peripheral vascular disease, cerebrovascular accident or transient ischemic attack (TIA), dementia, chronic obstructive pulmonary disease, connective tissue disease, peptic ulcer disease, liver disease, diabetes mellitus, hemiplegia, and moderate-to-severe chronic kidney disease were also searched using the ICD-10 codes.

### 2.3. Primary and Secondary Outcomes

The primary outcome was the comparison of the overall and specific cancer incidence by gender and by follow-up time interval between the HZ and non-HZ groups. The secondary outcome was the comparison of the incidence of specific cancers by follow-up time interval between the PHN (presence of PHN in the HZ group) and non-PHN groups (absence of PHN in the HZ group). PHN was defined as persistent chronic pain of >3 months after the diagnosis of HZ [29].

### 2.4. Statistical Analysis

After performing the 1:1 matching by age, sex, and CCI, differences in the proportions of characteristics between the HZ and non-HZ groups were compared by chi-square tests and presented as frequencies. Each patient was followed up from the cohort entry date to cancer diagnosis, death, or the end of the study (30 December 2015). Data were expressed per person-years of follow-up. We calculated the incidence rate per 1000 person-years as the number of cases divided by 1000 person-years, that is, the incidence rate = the number of cases/the sum of time spent in the study across all the participants × 1000. We used Cox proportional hazard regression models with robust standard errors to estimate the hazard ratios (HRs) for the two groups. The HRs were compared by setting the intervals at 1, 3, and 5 years. In addition, we used the log-rank tests to evaluate the difference in the risk of cancer between the two groups. The model was fitted using a SAS PHREG procedure (SAS Institute Inc., Cary, NC, USA). Schoenfeld residual plots were used to assess the proportionality of the hazards in the model. All the reported *p* values were two-sided, and *p* < 0.05 was considered statistically significant. Data manipulation and statistical analyses were conducted using SAS^®^ Version 9.4 (SAS Institute Inc., Cary, NC, USA).

## 3. Results

Table 1 shows the basic demographic characteristics and CCI of the study participants. There were more women than men in both groups, with the predominant age being the 40s and 50s. The CCI was mostly 0, 1, and 2 points.

### 3.1. Primary Outcomes

Table 2 shows the HRs for the risk of cancer between the HZ and matched non-HZ groups. The incidence rate in the HZ and non-HZ groups was 10.55 vs. 11.34 per 1000 person-years, respectively, with a significantly lower HR of 0.94 (95% confidence interval [CI] 0.90–0.97, *p* = 0.002) in the HZ group than in the non-HZ group. After the sex stratification, a significant HR for the risk of cancer of 0.93 (95% CI, 0.87–0.98, *p* = 0.015) in men and a non-significant HR in women of 0.94 (95% CI, 0.89–1.00, *p* = 0.052) were found. The statistically significant HRs for the risk of cancer at the 1-, 3-, and 5-year follow-up time interval were 0.85 (95% CI, 0.77–0.94, *p* = 0.002), 0.89 (95% CI, 0.84–0.94, *p* < 0.001), and 0.92 (95% CI, 0.88–0.96, *p* = 0.001), respectively (Table 2).

The HRs for the specific cancers between the HZ and non-HZ groups by gender and follow-up time intervals are shown in Table 3. The overall HRs for the risk of specific cancer were 0.41 (95% CI, 0.33–0.50, *p* < 0.001); 0.86 (95% CI, 0.81–0.91, *p* < 0.001); 0.87 (95% CI, 0.78–0.97, *p* = 0.014); 0.80 (95% CI, 0.73–0.87, *p* < 0.001); 1.20 (95% CI, 1.07–1.34, *p* = 0.001); and 1.66 (95% CI, 1.35–2.03, *p* < 0.001) for the malignant neoplasms of the lips, mouth, and pharynx; digestive system; respiratory system; unknown secondary and unspecified sites; thyroid gland and endocrine gland; and lymphoid and hematopoietic systems, respectively. Overall, there was no significant change in the HR by gender and follow-up time interval between the HZ and non-HZ groups.

**Figure 2:**
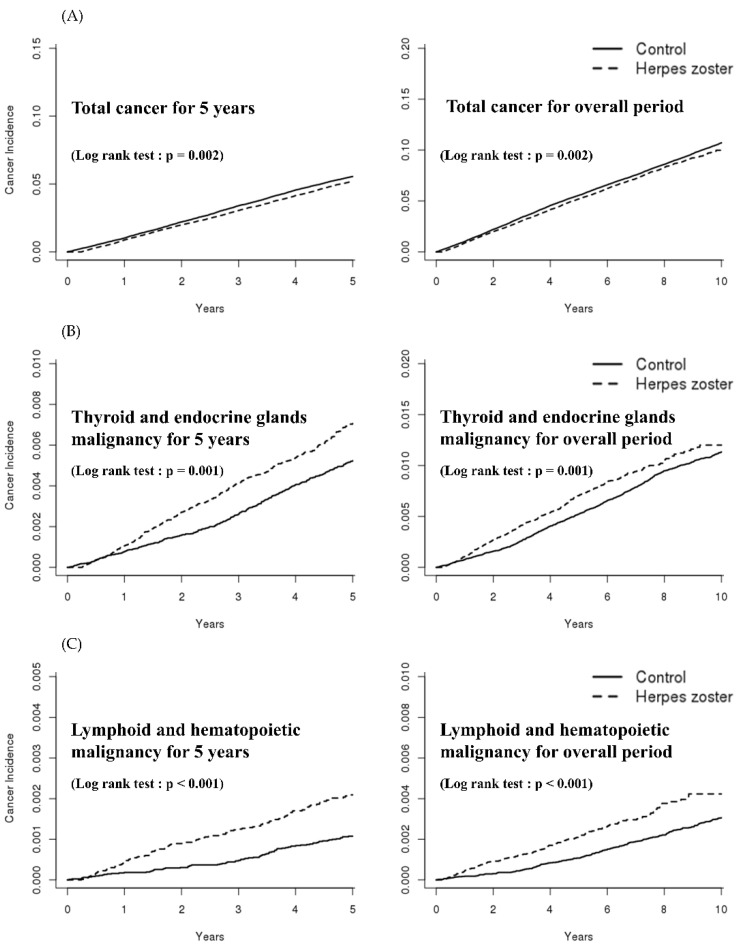

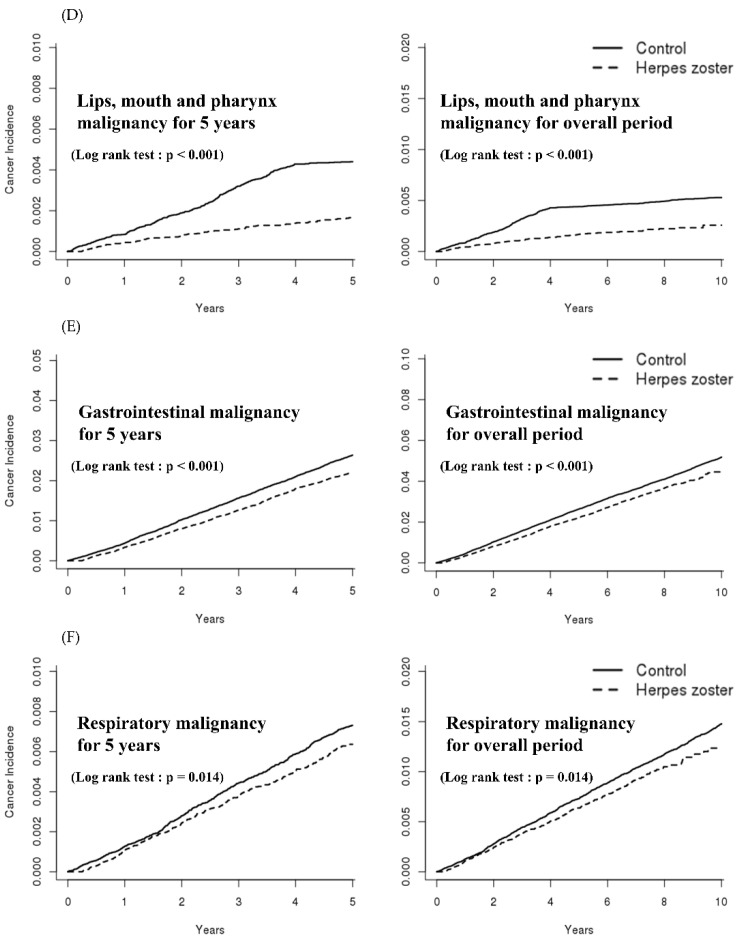
Kaplan–Meier (KM) curve for specific cancer in the HZ group and matched non-HZ group (Lt: for 5 years, Rt: overall). (**A**) KM curve for total cancer in the HZ and non-HZ groups (Lt: for 5 years, Rt: overall). (**B**) KM curve for thyroid and endocrine gland malignancies in the HZ and non-HZ groups (Lt: for 5 years, Rt: overall). (**C**) KM curve for lymphoid and hematopoietic malignancies in the HZ and non-HZ groups (Lt: for 5 years, Rt: overall). (**D**) KM curve for the lips, mouth, and pharynx malignancies in the HZ and non-HZ groups (Lt: for 5 years, Rt: overall). (**E**) KM curve for gastrointestinal malignancy in the HZ and non-HZ groups (Lt: for 5 years, Rt: overall). (**F**) KM curve for respiratory malignancy in the HZ and non-HZ groups (Lt: for 5 years, Rt: overall).

The Kaplan–Meier curve shows the specific cancer incidence between the HZ and matched non-HZ groups (Figure 2).

### 3.2. Secondary Outcomes

The HRs between the two groups (HZ with PHN vs. HZ without PHN groups) showing associations with specific cancer incidence by the follow-up time intervals are shown in Table 4. The overall HRs for the risk of specific cancer were 1.33 (95% CI, 1.20–1.47, *p* < 0.001); 1.65 (95% CI, 1.37–1.99, *p* < 0.001); 1.41 (95% CI, 1.21–1.63, *p* < 0.001); and 1.74 (95% CI, 1.27–2.39, *p* < 0.001) in malignant neoplasms of the digestive system; respiratory system; unknown secondary and unspecified sites; and lymphoid and hematopoietic systems, respectively (Table 4). There was no significant difference in HRs between the two groups during the 1-year follow-up period for the digestive system, unknown secondary and unspecified sites, and lymphoid and hematopoietic system neoplasms; however, all cancers’ risks showed significant differences with increasing follow-up periods.

## 4. Discussion

This was a large population-based retrospective study to identify the association between HZ and the risk of cancer. Our study demonstrated that HZ was associated with a decreased risk of overall cancers. However, when subdivided into specific cancers, the risks of certain cancers, such as oropharyngeal, gastrointestinal, and respiratory malignancies, were decreased, and the risk of cancer increased for hematological malignancies, and thyroid and other endocrine malignancies. In addition, PHN group patients showed a higher incidence of gastrointestinal, respiratory, and hematologic malignancies compared with HZ group patients without PHN. These results suggest that HZ and PHN were associated with the development of certain cancers.

There have been many case control studies on the association between HZ and the risk of cancer in Western and Eastern cohorts. The association between cancer and HZ has been recognized since 1955 [11], and until the early 2000s, four studies found no significant increase in cancer risk among HZ patients [10,16,20,30]. In 2004, Sorensen et al. found an increased risk of cancer after the first year of HZ diagnosis, and they also reported a particularly high risk for hematologic cancer in this group [13]. Recently, Chiu et al. suggested that HZ may be a marker of occult malignancy, particularly in lung cancer [22]. However, Parag et al. concluded that HZ was associated with a modest increase in the risk of a few cancers, particularly hematologic malignancies [17]. In 2017, a systematic review and meta-analysis of 46 previous studies reported that the relative risk for any cancer was 1.42 during the entire period of each study and 1.83 at one year of HZ diagnosis [15]. This meta-analysis supports the association between HZ and occult cancer, particularly hematologic malignancies, although the low absolute risk of cancer (0.7–1.1%) limits the clinical implications. There is still controversy on the association between HZ and the risk of cancer; therefore, this study may be clinically meaningful because it analyzed the association between HZ and the risk of cancer according to sex, follow-up time interval, specific cancers, and PHN.

Our results suggest that HZ might be an independent risk marker for some malignancies, such as lymphoid malignancy, but not for others. Furthermore, the risk of certain cancers increases further in patients with decreased immune function, such as those with PHN. In our view, this risk can be explained by three mechanisms. First, before diagnosis, hematologic malignancies are known to be present in a pre-clinical and undetectable form for ≥10 years [31,32,33], and HZ could be an early manifestation due to the immune system impairment provoked by the latent malignancy. Second, it is possible that VZV reactivation triggers immunological mechanisms, such as tissue antigen alteration or antigenic stimulation, thus leading to the development of malignancy [23]. This hypothesis is supported by the finding of different types of malignancies (lymphoma, pseudolymphoma, angiosarcoma, and Kaposi’s sarcoma) at the same site as a previous HZ infection [11,28,34,35]. Finally, cell-mediated immunity conferred by CD4+ T and natural killer cells has an important role in VZV reactivation [36,37] and the maintenance of immunologic surveillance in the control of malignancy [38,39]. Any dysfunction of such a mechanism may result in the emergence of both HZ and malignancy [19]. Some host factors can be associated with the development of lymphoid malignancy, including inherited genetic factors and other systemic diseases, such as autoimmune and infectious diseases. However, because CCI was considered when performing the 1:1 matching between the control and HZ groups, the impact of other systemic disease-related factors on the outcome is thought to be minimal. An interesting finding of our study was the association between HZ and digestive, and respiratory system malignancies. Unlike other previous studies [17,22], the HZ group had a higher risk of thyroid and other endocrine system malignancies and a lower risk of oropharynx, gastrointestinal, and respiratory system malignancies in our study. This may be the result of ours being a matched control study (by age, gender, and CCI) rather than a conventional cohort study or may actually reflect a true trend in the Korean race that is different from other races. Recently, Kim et al. reported that the incidence of liver, esophagus, and pharyngeal cancers is lower in the HZ group [40], and this result is consistent with our findings. In addition, the prevalence of digestive system cancers, such as stomach and esophageal cancers, differs significantly between the West and East, and this is known to be related to various factors, including diet and lifestyle [41,42]. These cultural and racial differences may have influenced our results, but the lack of prior research makes it difficult to draw conclusions. Therefore, there is a need for further comprehensive research on the relationship between racial differences and zoster.

This study has many strengths, including a well-defined source population; use of a 1:1 matched external control group in terms of age, sex, and CCI from the general population; and a large sample size. Nevertheless, our research has some limitations. First, miscoding and misclassification are potential biases in an established database that relies on physician-reported diagnoses. A similar skin disease may have been coded as HZ. It is also possible that a PHN code was entered for the use of neuropathic drugs in the insurance system. It is also possible that if an HZ vaccine was administered, even if HZ developed later, the patient may have been included in the untreated control group. Second, we did not account for residual unmeasured confounding factors, such as smoking, alcohol intake, and dietary habits, which may affect the immune status. Third, >99% of the Korean population is of Korean ethnicity; thus, the results of the present study may not be generalizable to other ethnic groups. Fourth, a spurious association between HZ and the risk of subsequent malignancy could occur if the HZ patients were more likely to be screened for cancer. However, such surveillance bias would have been minimal because there is limited public attention highlighting HZ as a risk factor for cancer in Korea.

## 5. Conclusions

In conclusion, our data demonstrated that HZ may be associated with the development of certain cancers such as lymphoid and endocrine cancers, but not all the cancers. Further, we also showed a further increased risk of certain cancers in the PHN group. Thus far, it remains unclear whether the early detection of HZ affects the prognosis of certain malignancies or whether the screening of HZ would be of help in the treatment of cancers. Further studies are needed to demonstrate the association between HZ and certain malignancies by investigating the pathophysiological mechanisms and preventive strategies in HZ patients.

## Figures and Tables

**Table 1:** Basic characteristics of the HZ group and matched non-HZ group.

Study Population	HZ	Non-HZ
Total	81,993	81,993
Sex (male)	33,003 (40.3%)	33,003 (40.3%)
Age	51.3 ± 15.7	51.3 ± 15.7
20~29 years	8213 (10.0%)	8213 (10.0%)
30~39 years	12,205 (14.9%)	12,205 (14.9%)
40~49 years	15,896 (19.4%)	15,896 (19.4%)
50~59 years	20,245 (24.7%)	20,245 (24.7%)
60~69 years	13,960 (17.0%)	13,960 (17.0%)
70~79 years	8630 (10.5%)	8630 (10.5%)
≥80 years	2844 (3.5%)	2844 (3.5%)
CCI		
0	37,556 (45.8%)	37,556 (45.8%)
1	22,258 (27.2%)	22,258 (27.2%)
2	11,335 (13.8%)	11,335 (13.8%)
3	5496 (6.7%)	5496 (6.7%)
4	2760 (3.4%)	2760 (3.4%)
5	2588 (3.2%)	2588 (3.2%)

Data are expressed as numbers (%) or means (standard deviations) as appropriate. The non-HZ group (one for every HZ patient) was matched with the HZ group in terms of age, sex, and Charlson comorbidity index. Abbreviations: HZ, herpes zoster; CCI, Charlson comorbidity index.

**Table 2:** The hazard ratios for developing cancers overall between the HZ group and the matched non-HZ group.

Variable	HZ	Non-HZ	HR	95% CI	*p*
Total	10.55	11.34	0.94	0.90–0.97	0.002
Sex					
Male	12.26	13.40	0.93	0.87–0.98	0.015
Female	9.42	9.98	0.94	0.89–1.00	0.052
Follow-up interval					
1 year	8.69	10.20	0.85	0.77–0.94	0.002
3 years	10.16	11.52	0.89	0.84–0.94	<0.001
5 years	10.43	11.43	0.92	0.88–0.96	0.001

Data are expressed as numbers. Incidence rate is per 1000 person-years. Abbreviations: HZ, herpes zoster; HR, hazard ratio; CI, confidence interval.

**Table 3:** The hazard ratios for specific cancers by gender and follow-up time interval between the HZ group and the matched non-HZ group.

Follow Up Period	Cancer Type	Total			Male			Female		
		HR	95% CI	*p*	HR	95% CI	*p*	HR	95% CI	*p*
Overall	Lips, mouth, and pharynx	0.41	0.33–0.50	<0.001	0.41	0.33–0.50	<0.001	0.41	0.33–0.50	<0.001
	Digestive system	0.86	0.81–0.91	<0.001	0.86	0.81–0.91	<0.001	0.86	0.81–0.91	<0.001
	Respiratory system	0.87	0.78–0.97	0.014	0.87	0.78–0.97	0.014	0.87	0.78–0.97	0.014
	Bone and articular cartilage	1.03	0.63–1.66	0.904	1.03	0.63–1.66	0.904	1.03	0.63–1.66	0.904
	Melanoma and skin	1.15	0.90–1.47	0.260	1.15	0.90–1.47	0.260	1.15	0.90–1.47	0.260
	Mesothelial and soft tissue	1.02	0.71–1.46	0.911	1.02	0.71–1.46	0.911	1.02	0.71–1.46	0.911
	Urinary tract	1.04	0.87–1.25	0.608	1.04	0.87–1.25	0.608	1.04	0.87–1.25	0.608
	Eye, brain, and CNS system	0.79	0.59–1.07	0.139	0.79	0.59–1.07	0.139	0.79	0.59–1.07	0.139
	Thyroid gland and endocrine gland	1.20	1.07–1.34	0.001	1.20	1.07–1.34	0.001	1.20	1.07–1.34	0.001
	Unknown secondary and unspecified sites	0.80	0.73–0.87	<0.001	0.80	0.73–0.87	<0.001	0.80	0.73–0.87	<0.001
	Lymphoid and hematopoietic system	1.66	1.35–2.03	<0.001	1.66	1.35–2.03	<0.001	1.66	1.35–2.03	<0.001
1 year	Lips, mouth, and pharynx	0.50	0.33–0.76	0.001	0.80	0.39–1.61	0.538	0.40	0.24–0.67	0.001
	Digestive system	0.76	0.65–0.90	0.001	0.85	0.68–1.06	0.172	0.68	0.54–0.86	0.001
	Respiratory system	0.84	0.63–1.12	0.256	0.90	0.61–1.32	0.606	0.77	0.49–1.20	0.257
	Bone and articular cartilage	0.87	0.26–2.86	0.827	0.52	0.09–2.84	0.451	1.59	0.26–9.44	0.607
	Melanoma and skin	0.98	0.49–1.95	0.958	1.20	0.43–3.32	0.715	0.82	0.33–2.11	0.690
	Mesothelial and soft tissue	1.82	0.53–6.26	0.338	3.19	0.33–3.10	0.310	1.37	0.35–6.21	0.678
	Urinary tract	0.95	0.59–1.53	0.858	0.92	0.51–1.63	0.780	1.04	0.40–2.41	0.922
	Eye, brain, and CNS system	1.13	0.51–2.49	0.749	0.70	0.19–2.48	0.584	1.56	0.57–4.42	0.394
	Thyroid gland and endocrine gland	1.36	0.98–1.89	0.065	1.05	0.36–2.99	0.926	1.40	0.90–1.98	0.057
	Unknown secondary and unspecified sites	0.78	0.60–1.00	0.056	0.69	0.47–1.02	0.065	0.85	0.60–1.19	0.359
	Lymphoid and hematopoietic system	2.44	1.33–4.48	0.004	1.30	0.61–2.80	0.491	7.00	2.09–23.61	0.002
3 years	Lips, mouth, and pharynx	0.35	0.27–0.45	<0.001	0.47	0.31–0.72	0.001	0.30	0.22–0.42	<0.001
	Digestive system	0.80	0.73–0.87	<0.001	0.83	0.73–0.93	0.003	0.76	0.67–0.87	<0.001
	Respiratory system	0.85	0.72–1.00	0.052	0.84	0.68–1.03	0.105	0.87	0.67–1.12	0.291
	Bone and articular cartilage	0.53	0.25–1.11	0.096	0.28	0.08–1.01	0.053	0.82	0.31–2.15	0.694
	Melanoma and skin	1.34	0.90–2.01	0.147	1.06	0.58–1.94	0.838	1.62	0.93–2.80	0.082
	Mesothelial and soft tissue	1.07	0.61–1.87	0.807	0.97	0.44–2.13	0.943	1.18	0.53–2.65	0.672
	Urinary tract	1.07	0.82–1.40	0.590	1.12	0.81–1.55	0.487	0.99	0.62–1.58	0.980
	Eye, brain, and CNS system	0.89	0.57–1.39	0.614	0.73	0.37–1.46	0.384	1.03	0.56–1.86	0.920
	Thyroid gland and endocrine gland	1.58	1.32–1.90	<0.001	1.79	1.02–3.12	0.041	1.56	1.29–1.88	<0.001
	Unknown secondary and unspecified sites	0.74	0.65–0.84	<0.001	0.70	0.58–0.85	<0.001	0.77	0.64–0.92	0.006
	Lymphoid and hematopoietic system	2.61	1.79–3.81	<0.001	2.14	1.28–3.57	0.004	3.31	1.88–5.82	<0.001
5 years	Lips, mouth, and pharynx	0.37	0.30–0.46	<0.001	0.55	0.38–0.80	0.002	0.30	0.23–0.40	<0.001
	Digestive system	0.83	0.77–0.89	<0.001	0.84	0.76–0.92	<0.001	0.82	0.74–0.91	<0.001
	Respiratory system	0.86	0.76–0.98	0.032	0.82	0.70–0.98	0.029	0.93	0.75–1.14	0.496
	Bone and articular cartilage	0.86	0.49–1.51	0.604	0.68	0.31–1.50	0.346	1.13	0.49–2.60	0.768
	Melanoma and skin	1.28	0.93–1.76	0.127	1.16	0.70–1.93	0.548	1.36	0.90–2.06	0.136
	Mesothelial and soft tissue	0.99	0.64–1.55	0.992	1.15	0.61–2.15	0.652	0.85	0.45–1.61	0.634
	Urinary tract	1.04	0.84–1.29	0.679	1.11	0.85–1.45	0.432	0.93	0.64–1.35	0.719
	Eye, brain, and CNS system	0.86	0.61–1.23	0.428	0.80	0.46–1.40	0.449	0.91	0.57–1.43	0.693
	Thyroid gland and endocrine gland	1.36	1.19–1.56	<0.001	1.32	0.90–1.93	0.153	1.37	1.18–1.59	<0.001
	Unknown secondary and unspecified sites	0.72	0.65–0.80	<0.001	0.73	0.63–0.84	<0.001	0.71	0.62–0.82	<0.001
	Lymphoid and hematopoietic system	2.00	1.52–2.63	<0.001	1.85	1.24–2.77	0.002	2.14	1.47–3.11	<0.001

Data are expressed as numbers. Abbreviations: HZ, herpes zoster; HR, hazard ratio; CI, confidence interval; CNS, central nervous system.

**Table 4:** The hazard ratios for specific cancers by follow-up time intervals between the PHN and non-PHN group (effect of PHN in HZ patients).

Cancer Type	Overall			Follow-Up at 1 year			Follow-Up at 3 Years			Follow-Up at 5 Years		
	HR	95% CI	*p*	HR	95% CI	*p*	HR	95% CI	*p*	HR	95% Cl	*p*
Lips, mouth, and pharynx	1.43	0.97–2.10	0.071	1.26	0.60–2.64	0.534	1.18	0.72–1.93	0.514	1.22	0.80–1.87	0.358
Digestive system	1.33	1.20–1.47	<0.001	1.20	0.92–1.57	0.169	1.38	1.19–1.59	<0.001	1.34	1.19–1.51	<0.001
Respiratory system	1.65	1.37–1.99	<0.001	1.67	1.07–2.62	0.023	1.71	1.33–2.21	<0.001	1.62	1.31–2.00	<0.001
Bone and articular cartilage	1.54	0.72–3.30	0.262	0.76	0.09–6.77	0.803	0.75	0.16–3.53	0.716	1.21	0.47–3.12	0.691
Melanoma and skin	1.50	0.96–2.19	0.079	1.37	0.48–3.95	0.556	1.13	0.61–2.09	0.693	1.11	0.67–1.86	0.681
Mesothelial and soft tissue	0.83	0.41–1.67	0.603	1.21	0.24–6.24	0.819	0.45	0.13–1.51	0.195	0.67	0.28–1.62	0.371
Urinary tract	1.18	0.86–1.61	0.303	0.81	0.35–1.88	0.628	1.35	0.89–2.05	0.160	1.27	0.89–1.81	0.182
Eye, brain, and CNS system	1.27	0.74–2.21	0.388	0.55	0.12–2.48	0.436	1.12	0.52–2.41	0.768	1.17	0.63–2.17	0.618
Thyroid gland and endocrine gland	1.05	0.86–1.28	0.638	0.85	0.50–1.43	0.542	0.96	0.73–1.25	0.739	1.06	0.85–1.32	0.594
Unknown secondary and unspecified sites	1.41	1.21–1.63	<0.001	1.15	0.75–1.76	0.516	1.36	1.10–1.70	0.005	1.41	1.19–1.69	<0.001
Lymphoid and hematopoietic system	1.74	1.27–2.39	<0.001	1.78	0.90–3.54	0.097	1.34	0.85–2.12	0.198	1.46	1.01–2.13	0.043

Data are expressed as numbers. Abbreviations: PHN, post herpetic neuralgia; HZ, herpes zoster; HR, hazard ratio; Cl, confidence interval; CNS, central nervous system.

## Data Availability

The dataset used and/or analyzed during the current study is available from the corresponding author on any reasonable request.
